# Transcription Factor SsSte12 Was Involved in Mycelium Growth and Development in *Sclerotinia sclerotiorum*

**DOI:** 10.3389/fmicb.2018.02476

**Published:** 2018-10-17

**Authors:** Tingtao Xu, Jingtao Li, Baodong Yu, Ling Liu, Xianghui Zhang, Jinliang Liu, Hongyu Pan, Yanhua Zhang

**Affiliations:** ^1^College of Plant Sciences, Jilin University, Changchun, China; ^2^College of Plant Health and Medicine, Qingdao Agricultural University, Qingdao, China; ^3^Department of Emergency of Xinmin, China-Japan Union Hospital of Jilin University, Changchun, China

**Keywords:** SsSte12, *Sclerotinia sclerotiorum*, development, appressoria, pathogenicity, SsMcm1, interaction

## Abstract

*Sclerotinia sclerotiorum* is a challenging agricultural pathogen for management, causing large global economic losses annually. The sclerotia and infection cushions are critical for its long-term survival and successful penetration on a wide spectrum of hosts. The mitogen-activated protein kinase (MAPK) cascades serve as central signaling complexes that are involved in various aspects of sclerotia development and infection. In this study, the putative downstream transcription factor of MAPK pathway, SsSte12, was analyzed in *S. sclerotiorum.* Silencing *SsSte12* in *S. sclerotiorum* resulted in phenotypes of delayed vegetative growth, reduced size of sclerotia, and fewer appressoria formation. Consequently, the *SsSte12* RNAi mutants showed attenuated pathogenicity on the host plants due to the defect compound appressorium. Yeast two-hybrid (Y2H) and bimolecular fluorescence complementation assays demonstrated that the SsSte12 interacts with SsMcm1. However, the *SsMcm1* expression is independent of the regulation of *SsSte12* as revealed by qRT-PCR analysis in *SsSte12* RNAi mutants. Together with high accumulation of *SsSte12* transcripts in the early development of *S. sclerotiorum*, our results demonstrated that SsSte12 function was essential in the vegetative mycelial growth, sclerotia development, appressoria formation and penetration-dependent pathogenicity. Moreover, the SsSte12-SsMcm1 interaction might play a critical role in the regulation of the genes encoding these traits in *S. sclerotiorum*.

## Introduction

*Sclerotinia sclerotiorum* (Lib.) de Bary is in the filamentous fungal genus *Scleroinia* in the family of Sclerotiniaceae in order Helotiales of the Ascomycota (Amselem et al., 2011). It is a devastating necrotrophic fungal plant pathogen that causes disease in >400 plant species (Boland and Hall, 1994). Over 60 names were used to refer to the plant disease caused by *S. sclerotiorum* including‘lettuce drop,’ ‘watery soft rot,’ ‘white mold,’ and ‘stem rot’ (Purdy, 1979). The difficulties associated with sclerotinia disease control can be partially attributed to the sclerotia development in the disease life cycle. Sclerotia can remain dormant and retain the viability of the pathogen for several years under harsh biological and physical environments including low temperature, microbially active soils, and dry environments; the results were documented for up to 8 years (Adams and Ayers, 1979). Mycelia from sclerotia could directly infect the plant tissues by forming infection cushions (Adams and Ayers, 1979) or enter the plant tissue through open stomata via deregulating the guard cells on the leaves (Guimarães and Stotz, 2004). The penetration of host cells starting from saprophytic mycelium is initiated via hyphal aggregations, known as infection cushions (compound appressorium) (Williamson et al., 2007). Due to the adaptations for long-term survival and pathogenicity on hosts via myceliogenic germination, controlling the plant diseases caused by *S. sclerotiorum* remains yet a challenge for modern agriculture (Bolton et al., 2006).

Regarding development, hardened, multicellular sclerotia was formed from the aggregation of vegetative hyphae enclosed by a melanized rind layer. The development of sclerotia involves several distinct stages (Li and Rollins, 2009) and is tightly regulated by many intrinsic genetic factors. For example, controlling the sclerotinia diseases by suppressing sclerotia formation could reduce new infections by interfering with the life cycle. Importantly, lost the capacity to produce normal sclerotia in these pathogens attenuate their virulence or the ability to cause disease (Rollins, 2003; Erental et al., 2007; Li et al., 2017). Understanding the relationship between sclerotia development and pathogenicity may provide insights into the genetic links between fungal development and pathogenicity.

The development of sclerotia is mediated by both PKA (protein kinase A)-dependent and PKA-independent processes. Mitogen-activated protein kinase (MAPK) play key roles in PKA-independent and cross-talking pathways during sclerotium development (Harel et al., 2006; Erental et al., 2008; Li and Rollins, 2010). MAPK is enzymatically activated by sequential phosphorylation events in response to various extracellular stimuli; also, it could phosphorylate the transcription factors and regulate gene expression (Erental et al., 2008). The activity of protein phosphatase types 2A and 2B has been recently shown to be dependent on MAPK (Smk1) and required for sclerotia development (Harel et al., 2006; Erental et al., 2007).

The MAPKs have also been shown to be essential for appressorium formation and infection of plant pathogenic fungi, as well as, in the regulation of sclerotia development (Zhao et al., 2007). In *Saccharomyces cerevisiae*, the transcription factor Ste12 could be activated by the Fus3/Kss1 MAPK cascade for regulating the mating processes; however, it requires several interacting components (Chou et al., 2006). In plant pathogenic fungi, no direct evidence showed whether the activity of Ste12 was regulated by the MAPK pathway (Schamber et al., 2010). Nevertheless, in *Neurospora crassa*, such a regulatory link has been presented as mutants defective in the MAPK and the Ste12 show similar phenotypes (Li et al., 2005). In *Botrytis cinerea*, mutants lacking Ste12 delayed the infection as a result of low penetration efficiency, and also, there was evidence for a regulatory link between the MAP kinase cascade and Ste12 (Schamber et al., 2010). In addition, Ste12 was required for the pathogenicity by regulating appressorium development and penetration in *Setosphaeria turcica* and rice blast fungus (Park et al., 2004; Gu et al., 2014).

In yeast, the Ste12 homeodomain binds either to pheromone response elements (PREs; TGAAACR) in mating gene promoters (Yuan and Fields, 1991) or filamentation response elements (FREs) on filamentous growth-specific target genes (Madhani and Fink, 1997) depending on the interaction partner. In addition, a key mechanism ensuring specificity between mating and invasive growth responses involves the selective interaction of Ste12 with different cofactors such as Mcm1, Far1, or Tec1 (Rispail and Di Pietro, 2010). Among these cofactors, the MADS-box transcription factor Mcm1, is conserved in filamentous fungi (Nolting and Pöggeler, 2006). However, whether Ste12-like proteins bind solely or as homo- or hetero-dimers of the *cis*-acting sequences in *S. sclerotiorum* is yet poorly understood.

In the present study, the *SsSte12* gene in *S. sclertiorum*, predicted to encode a Ste12 transcription factor, is orthologous to the *B. cinerea Ste12* and the rice blast fungus *Mst12* (Ste12 homolog) genes, which regulate the penetration peg formation during plant infection (Park et al., 2004; Schamber et al., 2010). Herein, we functionally characterized *SsSte12* by gene knockdown strategy using RNA interference (RNAi) and focused on the hyphae growth, sclerotia development, compound appressorium formation, and virulence on hosts. In addition, we used Y2H and bimolecular fluorescence complementation (BiFC) assays to identify the interaction of SsSte12 with the MADS-box transcription factor, SsMcm1, which was previously named SsMADS (orthologous to yeast MCM1) and reported to be involved in the growth and virulence in *S. sclerotiorum* (Qu et al., 2014).

## Materials and Methods

### Fungal Strains, Culture Conditions, and Plant Materials

The wild-type *S. sclerotiorum* isolate UF1 was purified from petunia (University of Florida, unpublished). The cultures were routinely grown on potato dextrose agar (PDA) at room temperature. The RNAi mutants were cultured on PDA or regeneration medium (RM) (Li et al., 2017) supplemented with 100 μg/mL hygromycin B (Roche, Indianapolis, IN, United States). Hyphae stocks were maintained as desiccated mycelia-colonized filter paper or as dry sclerotia at −20°C. For the apothecia, mature sclerotium was produced using autoclaved smashed potato medium (SPM) with 1.5% agar and grown in the laboratory at a temperature 22–25°C. Apothecia were induced from the SPM culture-derived sclerotia according to the method described previously (Li and Rollins, 2010).

Bush bean and tomato were grown under fluorescent lighting at a temperature range 22–25°C in the laboratory. The seeds from tomato and bush bean were planted in potting soil mix (vermiculite: humus = 1:2) and grown in 4″ plastic pots.

### Cloning and Sequence Analysis of *SsSte12* Gene

Genomic DNA was isolated from *S. sclerotiorum* based on the *A. nidulans* DNA preparation protocol (Yelton et al., 1984). The *SsSte12* gene was cloned from the *S. sclerotiorum* genomic DNA using the primers (*SsSte12*-F and *SsSte12*-R; **Supplementary Table S1**). The sequence of *SsSte12* gene is annotated as locus SS1G_07136 (GenBank Accession No. NW_001820827.1) of the *S. sclerotiorum* 1980 UF-70 genome (Derbyshire et al., 2017). The BLASTX program on NCBI^1^ was used to search for *SsSte12* homologs in other fungi species. The phylogenetic tree was generated using the neighbor-joining method (NJ) in MEGA5 (Tamura et al., 2011). SmartBLAST on NCBI^2^ was used to complete the basic analysis of the SsSte12 protein sequence against the database for locating highly conserved regions and identifying the missing regions (Zou et al., 2017).

### Generation of SsSte12 Silencing Constructs and Genetic Manipulation of *S. sclerotiorum*

The *SsSte12* gene silencing vector construction strategy was described previously (Qu et al., 2014). Two independent target fragments for *SsSte12* gene silencing were amplified and integrated into the pSilent-Dual1 plasmid individually as described previously (Qu et al., 2014). The primers used to clone target 2 (498–786 bp) and target 3 (861–1336 bp) of the *SsSte12* gene from the cDNA (**Supplementary Table S1**) (pSD2-*SsSte12*-F, pSD2-*SsSte12*-R, pSD3-*SsSte12*-F, and pSD3-SsSte12-R). Both target fragments were cloned into pSilent-Dual1 vectors and termed as pSD2 and pSD3, respectively. All the digestions, gel electrophoresis, DNA purifications, and ligations were performed according to the manufacturer’s instructions. The *S. sclerotiorum* protoplasts transformation and screening by 100 μg/mL geneticin selection on RM medium was carried out as described previously (Rollins, 2003). The verification of the *SsSte12*-silenced transformants was conducted after three hyphal tip transfers for purification (Li et al., 2017). The PCR using primers (G418-F and G418-R) of the geneticin-resistant gene was used to identify the *SsSte12*-silenced transformants. In addition, the fungal transformation of empty vector pSilent-Dual1 (pSD-EV) was used as control in functional studies.

### Quantitative RT-PCR Assay

Quantitative real-time PCR (qRT-PCR) analysis of *SsSte12* and *SsMcm1* transcript accumulation in wild-type tissue and *SsSte12*-silenced transformants was performed as defined previously (Fan et al., 2017). Total RNA was extracted from lyophilized mycelia tissue on cellophane PDA plates using TRIzol (Invitrogen, Carlsbad, CA, United States) according to the standard procedures. Total RNA, 2 μg, was used for reverse transcription with PrimeScript^TM^ RT reagent Kit (TaKaRa, Foster City, CA, United States). The cDNA was diluted 20-fold, and the quantitative expression assays were performed using the TaKaRa SYBR^®^ Green Reagent Kit as described previously (Li et al., 2017) and the relative quantification method (2^−ΔΔCt^) was used to analyze the data (Li et al., 2016). The qRT-PCR experiments were repeated three times, and data was normalized based on the housekeeping gene *Actin* (SS1G_08733). Primer pairs (qRT-*SsSte12*-F, qRT-*SsSte12*-R, qRT-*SsMcm1*-F, and qRT-*SsMcm1*-R) used for qRT-PCR are listed in **Supplementary Table S1**.

### Morphological Characterization

Control (wild-type and pSD-EV) and *SsSte2*-silenced strains were cultured on PDA medium in 9-cm Petri dishes at room temperature. Hyphal growth and sclerotia development phenotypes were recorded with camera at 6 and 15 days after inoculation (DAI). Mature sclerotia from each strain were collected and photographed by 15 DAI. Dry weights and sizes of these sclerotia were determined to access the sclerotia development. The hyphae radial growth was measured at different time points (0, 12, 24, 36, 48, and 60 h) after inoculation. For hyphal morphology, fresh PDA-colonized agar plug (5 mm in diameter) was placed on a glass slide (SAIL BRAND) and was placed in a box at room temperature for 24 h, which was used for subsequent observations under optical microscope (BA310Met-T, Xiamen, China). The compound appressoria were produced on a glass slide using agar plugs with growing hyphal tips in a moisture chamber for 2 days. These were later observed using a stereoscopic zoom microscope Nikon SMZ1500 (Nikon, Japan). To quantify the numbers of the pigmented compound appressoria, Image J software^3^ was used to conduct the particle analysis.

### Pathogenicity Assay

Freshly collected bush bean and tomato leaflets were inoculated with PDA-colonized agar plugs taken from the edge of 1 to 2-day-old cultures at room temperature. Three leaflets were inoculated with each strain, and the experiment was repeated three times. The differences in timing and extent of symptom development were recorded with a digital camera. The lesion area was measured by Image J software.

### Yeast Two-Hybrid (Y2H) and Bimolecular Fluorescence Complementation (BiFC) Assays

The interaction between SsSte12 and SsMcm1 was detected with Matchmaker Gold Y2H system (Clontech, Japan) and BiFC system as described previously (Wang et al., 2017). For Y2H assay, both SsSte12 and SsMcm1 (SS1G_05588) coding regions were cloned into pGBKT7 and pGADT7 for reciprocal protein–protein interaction confirmation, respectively. The resulting constructs were co-transformed into Y2H strain Y187 with different combinations according to the manufacturer’s instructions. The transformed yeast cells were plated on SD-Leu-Trp and were identified by PCR. Subsequently, the positive transformants were grown on the SD-Leu-Trp-His-Ade medium supplemented with X-Gal according to the yeast protocol handbook (Clontech, Japan).

For the BiFC assay, the coding regions of SsSte12 and SsMcm1 were fused into cYFP (pSAT4-cEYFP-N1) and nYFP(pSAT4-nEYFP-N1) vectors, respectively. The resulting constructs were used for *Arabidopsis* protoplast transformation via PEG/Ca^2+^-mediated method as described previously (Yoo et al., 2007). The fluorescent signal and localization of SsSte12 and SsMcm1 fusion proteins were detected using a confocal laser scanning microscope (Nikon ECLIPSE Ts2R, Melville, NY, United States). Excitation wavelength was 514 nm and detection rage of emission wavelength was 520–550 nm.

### Digital Gene Expression of *SsSte12* and *SsMcm1*

Recently, we profiled gene expression patterns in *S. sclerotiorum* from three different developmental stages (hyphae, sclerotia, and apothecia) through RNA-seq approach in our laboratory (unpublished data). Fresh hyphae tissue was collected from 3 days culture on PDA covered with cellophane. The fresh colonized agar plugs (5-mm diameter) was place on the cellophane and incubated in a humidity chamber for 3 days. For the sclerotia collection, the cultures were routinely grown on sterile SPM at room temperature (22 to 25°C). Mature sclerotia were produced on SPM and collected. Apothecia were induced from SPM culture-derived sclerotia using the method described previously (Li and Rollins, 2010).

The transcript abundance of *SsSte12* and *SsMcm1* was quantified using the fragments per kilobase of exon per million mapped fragments (FPKM) method as described previously (Yuan et al., 2013). This method could eliminate the influence of different gene lengths and sequencing levels while estimating the gene expression (Zhang et al., 2015). To identify transcripts of SsSte12 and SsMcm1 that were specifically expressed in different tissues, FPKM values were compared among samples, and transcripts with FPKM > 3 in a single tissue were selected. The FPKM means were generated from three technical replicate samples for each developmental stage. The FPKM values of housekeeping genes histones *H3* (SS1G_09608.3) and *H2A* (SS1G_02052) were used as endogenous control.

### Statistical Analysis

All statistical analyses were performed by Tukey’s HSD (Honestly Significant Difference) test using PASW Statistics 18 (SPSS, Inc., Chicago, IL, United States) and statistical comparisons using the one-way ANOVA. All the graphs were exported by GraphPad Prism 6 software (La Jolla, CA, United States).

## Results

### Identification and Analysis of the *SsSte12* Gene

**FIGURE 1:**
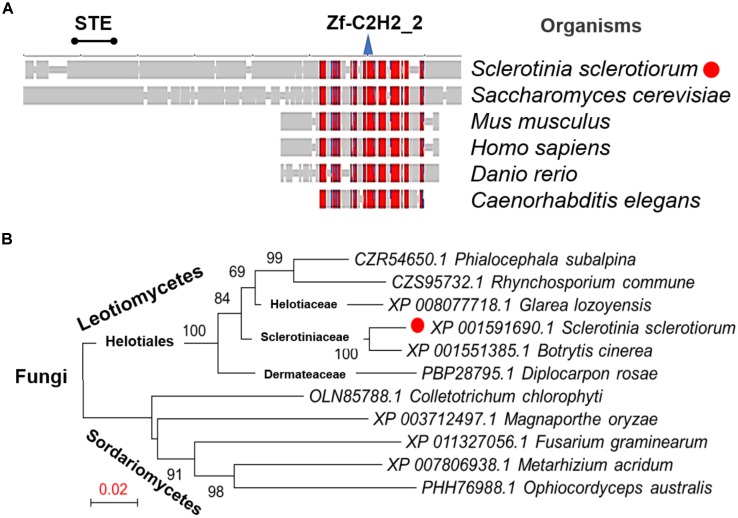
Conservation and phylogenetic analysis of Ste12 proteins. **(A)** SmartBLAST was used to locate the regions of high sequence conservation in a graphical view among different model organisms. STE motif and zinc-finger C_2_H_2_ motif were marked on the top of the graph. **(B)** Phylogenetic analysis of Ste12 proteins in *Sclerotinia sclerotiorum* and other fungi. The phylogenetic tree was generated by NJ method, and bootstrap support values of 1,000 replicates are given above the branches.

From the genome of *S. sclerotiorum* (Amselem et al., 2011; Derbyshire et al., 2017), hypothetical protein SS1G_07136 are annotated in the GenBank with the function of the transcription factor SteA. The orthologous relationship of the factor to *B. cinerea* Ste12 (GenBank Accession No. ACJ06644.1) and the *Marssonina brunnea* Ste12 protein (GenBank Accession No. XP_007290289.1) was determined based on best blastx results (E-value: 0.0). This protein was then termed as SsSte12 in *S. sclerotiorum*, which was predicted to contain an STE domain at the N terminus and a C2HC zinc finger domain at the C terminus by SmartBLAST (**Figure 1A**). The SmartBLAST analysis result displayed a wide taxonomic diversity in fungi (*S. sclerotiorum* and *S. cerevisiae*) and animal species (*Mus musculus*, *Homo sapiens*, *Danio rerio*, and *Caenorhabditis elegans*). The N-terminal STE motif of SsSte12 was not found in animal species in our analysis, while the C-terminal Zn-C_2_H_2_ conserved sequence presents in all the five matches (**Figure 1A**). Meanwhile, phylogenetic analysis demonstrated that Ste12 sequences from *S. sclerotiorum* and *B. cinerea* form a monophyletic group Sclerotiniaceae with a bootstrap of 100% (**Figure 1B**). The SsSte12 is also highly homologous to the Ste12 proteins from Helotiaceae and Dermateaceae within the clade of Leotiomycetes fungus (**Figure 1B**).

### Characterization of *SsSte12*-Silenced Mutants

**FIGURE 2:**
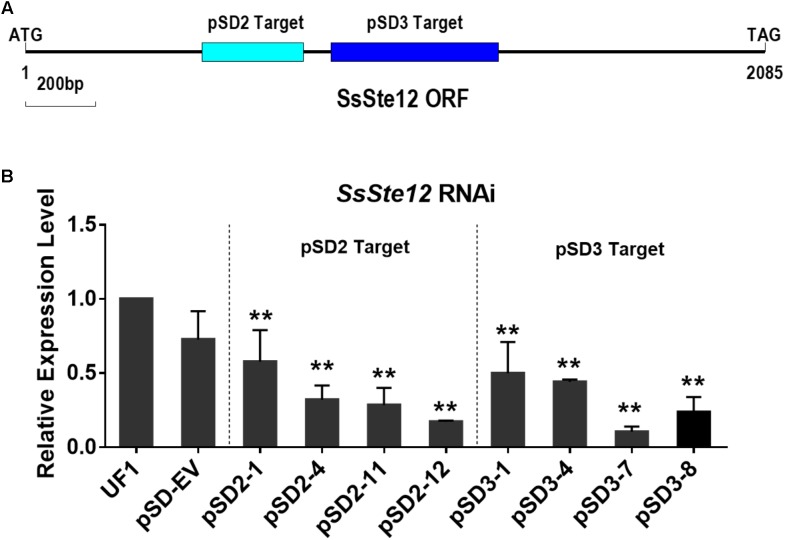
Generation of *SsSte12* silencing strains. **(A)** Generation of *SsSte12* silencing constructs. Two fragement he *SsSte12* in gene coding regions were used to generate the knockdown mutants pSD2 and pSD3 by gene silencing strategy. **(B)** *SsSte12* expression levels in wild-type strain UF1, empty vector transformant pSD-EV and silenced mutants (pSD2-1, pSD2-4, pSD2-11, and pSD2-12; pSD3-1, pSD3-4, pSD3-7, and pSD3-8). qRT-PCR was performed to profile the *SsSte12* expression. Gene expression values are normalized relative to the constitutively expressed *histone* gene. Values are the means ± SD (*n* = 3; ^∗∗^*P* < 0.01; one-way ANOVA).

To assess the function of SsSte12 in *S. sclerotiorum*, we silenced the *SsSte12* gene using RNAi. Two target sites (pSD2 and pSD3) were used to generate the *SsSte12* silencing mutants (**Figure 2A**). *SsSte12*-silenced transformants were selected on RM medium with 100 μg/mL geneticin and four PCR-positive transformants for each target were purified by three rounds of hyphal tip transfer.

**FIGURE 3:**
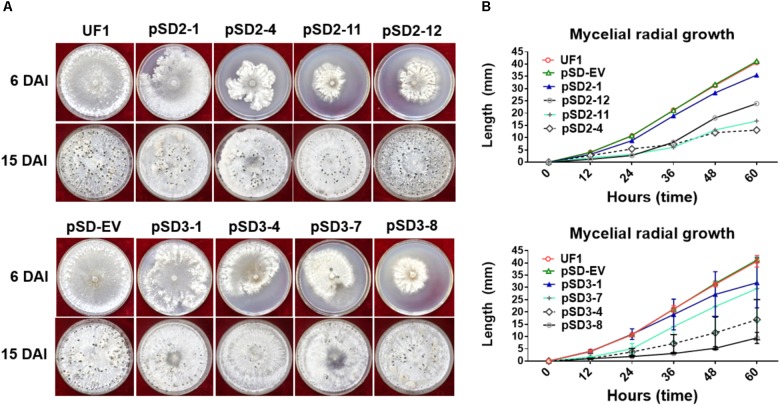
Morphological observations of UF1 (WT) and silencing strains. **(A)** Growth and sclerotium development phenotypes of the controls and RNAi mutants. UF1, pSD-EV, the four target-2 strains (pSD2-1, 4, 11, and 12), and four target-3 strains (pSD3-1, 4, 7, and 8) were on 9-cm diameter potato medium plates at 25°C for 6 and 15 days. **(B)** Growth rates of the controls and RNAi mutants. Mycelial radial growth was measured at 0, 12, 24, 36, 48, and 60 h post-inoculation. Values are expressed as means ± SD. Means were generated from three independent experiments.

**FIGURE 4:**
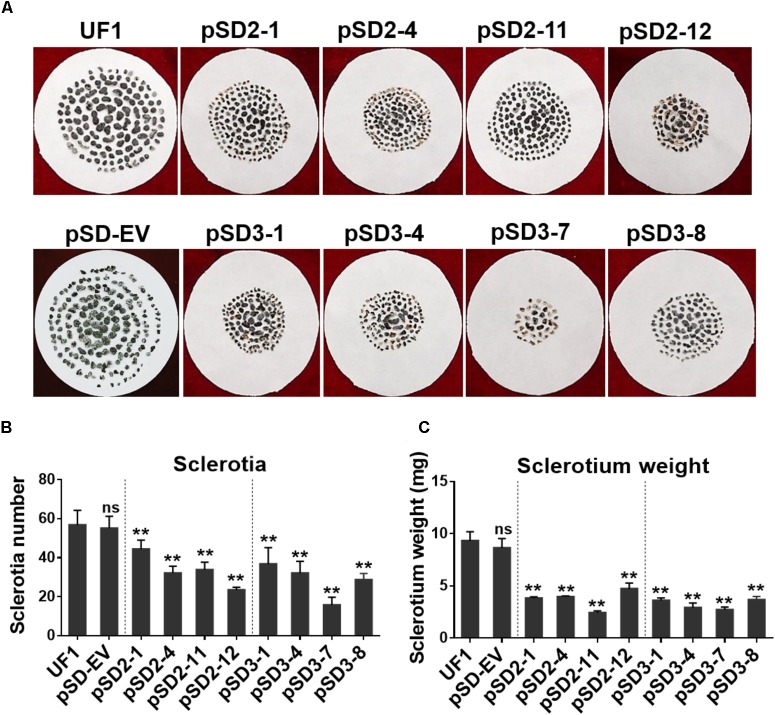
Effect of *SsSte12* silencing on sclerotium development. **(A)** Sclerotium morphology of the UF1 (WT) and silent mutants. Sclerotium was collected from 9-cm diameter potato medium at 15 days after inoculation. **(B,C)** Comparison of sclerotia production and dry weight among wild-type and silent mutants. Means were generated from three independent experiments (*n* = 3; ^∗^*P* < 0.05; ^∗∗^*P* < 0.01; one-way ANOVA).

**FIGURE 5:**
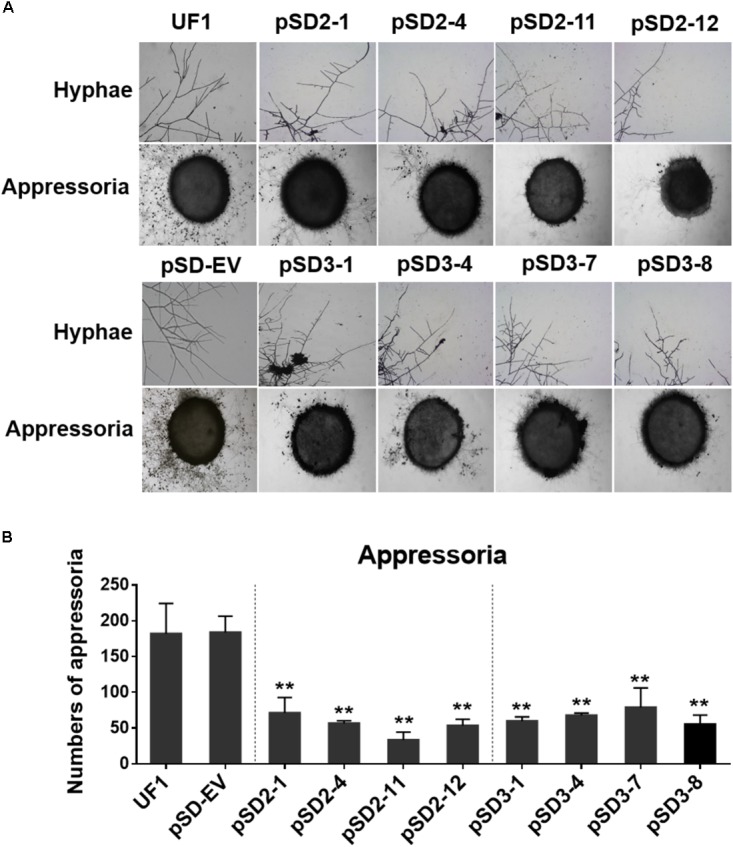
Effect of *SsSte12*-silenced on hyphal phenotypes and compound appressoria. **(A)** Morphological features of hyphae were observed by bright field light microscopy by 24 h after inoculation (HAI). Pigmented compound appressoria were observed on glass slide 2 days after inoculation (DAI) using mycelia plugs with 5-mm diameter. Images were captured by light microscopy. **(B)** The quantity of compound appressorium displayed a significant difference between the UF1 and silent mutants (*n* = 3; ^∗∗^*P* < 0.01).

*SsSte12* RNAi events were determined by qRT-PCR (**Figure 2B**). And four mutants with reduced transcripts of the SsSte12 for each target form a total of 32 mutants (15 mutants for pSD2 and 17 mutants for pSD3) were obtained. The relative expression level of *SsSte12* in silencing transformants demonstrated the homokaryotic gene silencing status of the *SsSte12* RNAi mutants. The transcript of *SsSte12* was detected at a moderate-to-low level in vegetative hyphae, indicating an efficient silencing of the *SsSte12* gene (**Figure 2B**). The *SsSte12* transcripts of pSD2 transformants (pSD2-1, pSD2-4, pSD2-11, and pSD2-12) and pSD3 transformants (pSD3-1, pSD3-4, pSD3-7, and pSD3-8) were significantly reduced as compared to the wild-type strain UF1. We also detected the *SsSte12* transcripts in the empty vector transformant (pSD-EV) and the results suggested that there is no significant transcript accumulation difference between the wild-type UF1 and pSD-EV (**Figure 2B**). At the same time, pSD-EV strain showed similar developmental phenotypes to UF1 (**Figures 3**–**5**), which indicated that the vector does not have negative effect on *S. sclerotiorum* normal development. Together, these results suggested that the knockdown of *SsSte12* by RNAi was efficient, and all these RNAi strains could be used for further morphological analysis.

### Silencing *SsSte12* Affects Hyphal Growth and Sclerotial Development

To investigate the role of SsSte12 in development, we compared the hyphal growth and sclerotial development in both control and silencing strains in normal growth conditions (**Figure 3A**). We observed obvious reduced hyphal growth and condensed colony at 6 DAI and 15 DAI in the RNAi strains; however, reduced levels were varied among different *SsSte12*-silenced strains, which is somewhat consistent to the silencing efficiency in each strain (**Figure 2B**). We also recorded the mycelium radial growth every 12 h to assess the role of *SsSte12* in hyphae growth. The *SsSte12*-silenced strains exhibited slower hyphal growth rates than the control strains (**Figure 3B**). Among them, pSD2-12, pSD2-4, pSD2-11, pSD3-4, and pSD3-8 strains displayed a substantially slow hyphal growth phenotype (**Figure 3B**).

When the cultures were observed following 15 days of cultivation on PDA media, the RNAi mutants exhibited abnormal sclerotia development over the entire colony surface (**Figure 3A**). In order to confirm this phenotype, mature sclerotia were collected from the plates. While *SsSte12*-silenced strains successfully formed sclerotia, the number per plate and average mass differed from those produced by the wild-type (**Figure 4**). *SsSte12*-silenced strains (both of pSD2 and pSD3) developed more small-sized sclerotia than wild-type; also, fewer sclerotia were formed by the pSD3 RNAi mutants on an average (**Figures 4A,B**). The sclerotia produced by RNAi strains were also less massive (**Figure 4C**) accounting for the reduced total sclerotia dry weight as compared to the UF1.

These results demonstrated that *SsSte12* functions in hyphal growth and sclerotial development in *S. sclerotiorum*.

### Silencing *SsSte12* Affects the Hyphae Structure and Compound Appressoria Quantity

A dramatic hyphal growth phenotype was observed in the *SsSte12*-silenced strains on the media. Next, we further investigated the hyphal tip morphology under light microscopy by 24 HAI. The microscope observations indicated that the RNAi mutants had perpendicular hyphal branching patterns (**Figure 5A**).

To determine whether *SsSte12* influences other multicellular developmental stages of *S. sclerotiorum*, we also examined the development of compound appressoria. The wild-type UF1 strain rapidly differentiated into abundant pigmented compound appressoria from vegetative hyphae by 2 DAI. Conversely, less compound appressoria were differentiated by the RNAi strains (**Figure 5A**). The number of compound appressoria produced by per 5 mm plug was calculated by Image J, which confirmed the significant differences of appressoria production between the wild-types and RNAi mutants (**Figure 5B**).

These data revealed that *SsSte12* was essential for hyphal morphology structure and compound appressoria quantity, but possibly not for appressorium formation.

### Silencing *SsSte12* Attenuates the Penetration-Dependent Pathogenicity

**FIGURE 6:**
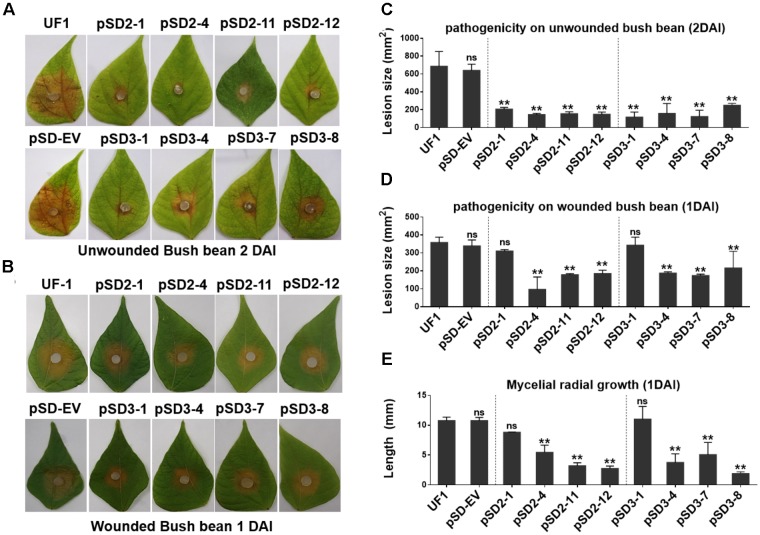
Silencing *SsSte12* attenuated the pathogenicity on hosts. **(A)** Symptom development on detached unwounded bush bean leaves was photographed by 2 DAI using mycelia-colonized PDA agar plugs. **(B)** Symptom development on detached wounded bush bean leaves was photographed by 1 DAI using mycelia-colonized PDA agar plugs. **(C,D)** Quantity of lesions (2 DAI pr 1 DAI) were calculated by Image J software (*n* = 3; ^∗∗^*P* < 0.01). **(E)** Mycelial radial growth was measured at 1 DAI. Values are expressed as means ± SD. Means were generated from three independent experiments.

To determine whether the compound appressorium deficiency of *SsSte12* caused a virulence deficiency in the hosts, tissue infection assays were performed (**Figure 6**). The wild-type and RNAi mutants were inoculated on detached unwounded bush bean and tomato leaves. By 2 DAI, the wild-type infected bush bean leaflets developed severe lesions; on the other hand, the RNAi mutants produced limited lesions surrounded by green tissue (**Figure 6A**). The lesion size was estimated by Image J, which also confirmed significant differences of virulence between the UF1 and *SsSte12* mutants (**Figure 6C**). The similar infection pattern was also observed by 2 DAI on another host plant, tomato, when different strains were inoculated on the detached leaflets (**Supplementary Figure S1**). We then inoculated the wounded bush bean leaves to clarify the effect of *SsSte12* on appressorium formation and pathogenicity. Both the wild-type and RNAi strains produced obvious lesions by 1 DAI, and no difference was observed with respect to virulence among wild-type, pSD2-1, and pSD3-1 (**Figures 6B,D**). The other RNAi mutants produced less severe lesions. Notely, the size of produced lesions on the wounded bush beans were quite consistant with the mycelial radial growth (**Figure 6E**) on PDA medium by 1 DAI. These phenotypes demonstrated that SsSte12 was partially involved in the penetration-dependent pathogenicity of *S. sclerotiorum*, and the virulence of the *SsSte12*-silenced mutants was also impaired on unwounded hosts due to the attenuated invasive mycelial growth on wounded hosts.

### Prediction and Identification of SsSte12 and SsMcm1 Interaction

To further analyze the function of SsSte12, we first predicted the interacting proteins of Ste12 by STRING database using *S. cerevisiae* as the model organism. After executing the search, five candidate proteins were obtained when reducing nodes and the functional linkage network was generated using the default parameters (**Supplementary Figure S2A**). This completely functional network encompassed the co-occurrence patterns across the genomes of dense interconnection genes that could be identified based on the similarities in different organisms, especially *S. sclerotiorum* (**Supplementary Figure S2B**). KSS1, FUS3, and MCM1 were largely conserved proteins, indicating their existence in *S. sclerotiorum*. Among these proteins, the function of MCM1, also termed as SsMcm1 in *S. sclerotiorum*, has been described in our previous study (Qu et al., 2014).

**FIGURE 7:**
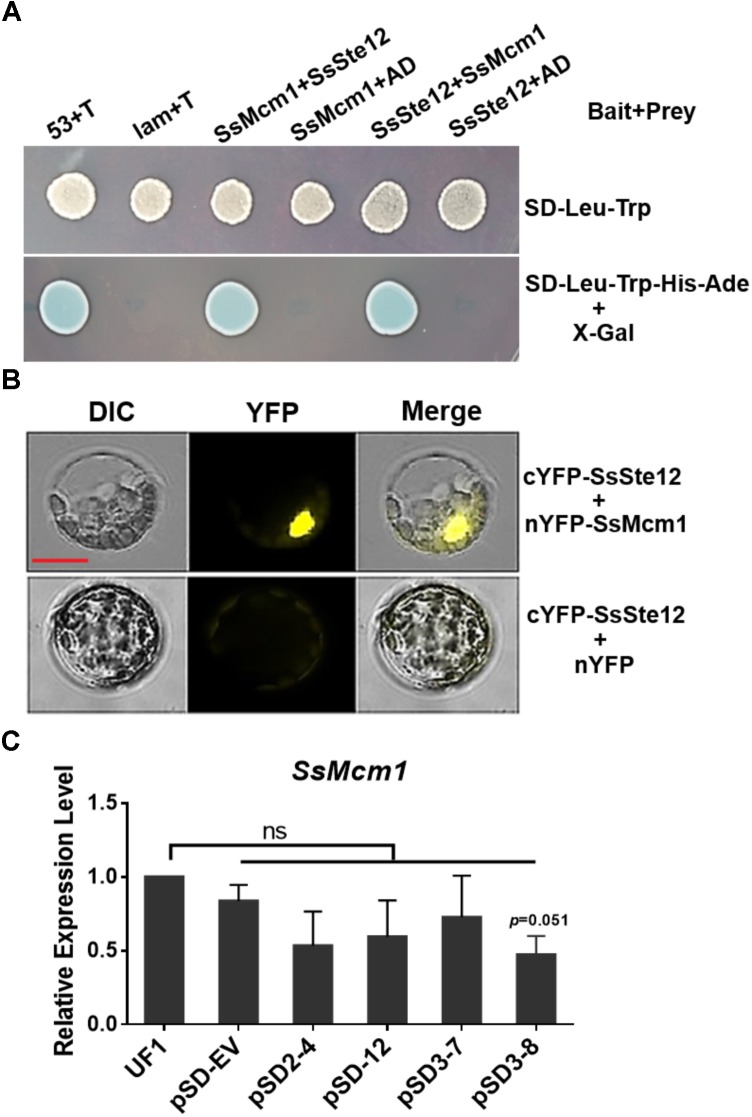
Interaction of SsSte12 and SsMcm1 proteins. **(A)** The specific interaction of the SsSte12 protein with SsMcm1 protein was revealed by a Y2H assay. The yeast cells were grown on SD-Leu-Trp or SD-Leu-Trp-His-Ade media with X-Gal. The yeast transformant cells with different plasmid pairs (bait + prey): 53 + T, pGBKT7-53, and pGADT7-T, which encoded two interacting proteins as the positive control (Clontech); Lam + T, pGBKT7-Lam, and pGADT7-T, served as the negative control; SsMcm1 + SsSte12, pGBKT7-SsMcm1, and pGADT7-SsSte12; SsMcm1 + AD, pGBKT7-SsMcm1, and pGADT7; SsSte12 + SsMcm1, pGBKT7-SsSte12, and pGADT7-SsMcm1; SsSte12 + AD, pGBKT7-SsSte12, and pGADT7. **(B)** BiFC demonstrated the interaction of the SsSte12 protein with SsMcm1 protein. BiFC signal was also clearly detected in the nucleus. Images were captured under a fluorescence microscope 14 h after transient expression in *Arabidopsis* protoplast. Scale bars = 10 μm. **(C)** qRT-PCR of *SsMcm1* expression level response in wild-type and *SsSte12*-silenced mutants. The constitutively expressed gene, *Actin*, was used as an internal control. Values are the means ± SD (*n* = 3; ns, no significant differences; one-way ANOVA).

To confirm the interaction between SsMcm1 and SsSte12, we performed Y2H and BiFC assay. The representative results of the SsMcm1-SsSte12 interaction identified in the Y2H assay are shown in **Figure 7A**. The protein–protein interaction was also confirmed by BiFC (**Figure 7B**). In BiFC, the *Arabidopsis* protoplast cells transformed with the constructs cYFP-sSte12 + nYFP-SsMcm1 generated a strong YFP signal in the nuclei, whereas no YFP signal was observed in those cells transformed with cYFP-SsSte12 + nYFP. Thus, these results indicated that SsSte12 interacted with SsMcm1.

To determine whether *SsMcm1* was regulated by SsSte12, we examined the *SsMcm1* transcript accumulation in four *SsSte12* RANi mutants by qRT-PCR. The expression profile did not reveal any no significant difference in the expression of *SsMcm1* in RNAi mutants, indicating that *SsMcm1* was beyond the regulation of a *SsSte12* pathway at transcriptional level (**Figure 7C**).

### Developmental Expression Profiling of *SsSte12* and *SsMcm1* in *S. sclerotiorum*

**FIGURE 8:**
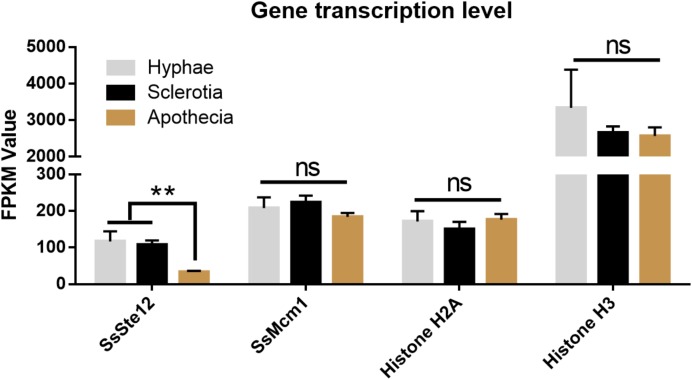
Differential expression of four genes (*SsSte12*, *SsMcm1*, *SsHisoneH2A*, and *SsHisoneH3*) at three developmental stages of *S. sclerotiorum*. Gene expression level was evaluated by the FPKM value. The *SsSte12* gene was significantly and differentially expressed as compared to the hyphae and sclerotia development stage (*n* = 3; ^∗∗^*P* < 0.01).

To detect whether *SsSte12* and *SsMcm1* transcription expression varies among different growth and development stages of *S. sclerotiorum*, we performed digital gene expression (DGE) analysis based on FPKM values obtained from the transcriptomes in three developmental stages (**Figure 8**). Only *SsSte12* showed a differential expression among the three stages (FPKM values decrease from 117.23 to 33.4), indicating a potential role of *SsSte12* in the early development of *S. sclerotiorum*. The *SsSte12* gene transcript accumulation during apothecium development was relatively lower than that during the vegetative mycelial growth and sclerotia development. Besides, the apothecium development was not defective in RNAi mutants (data not shown).

*SsMcm1* was expressed at the higher level (FPKM values ranged from 184.29 to 223.76) than *SsSte12*; however, no significant difference was found among these three developmental stages, indicating that *SsMcm1* may play a crucial role in both early development and apothecia development. Similar to *SsMcm1*, *Histone H2A* was expressed among all the three developmental stages (FPKM values ranged from 150.67 to 176.75), albeit slightly lower than that of *SsMcm1*. Moreover, *Histone H3* (FPKM values ranged from 2563.93 to 3337.34) displayed the highest expression level at all the three stages, exhibiting its predominant role as a housekeeping gene used as a control.

## Discussion

The sclerotium and infection cushions (compound appressorium) are critical for *S. sclerotiorum* long-term survival and successful penetration in wide spectrum of hosts. The MAPK pathways serve as central signaling complexes that are involved in various aspects of sclerotia development and infection in plant pathogenic fungi (Harel et al., 2006; Zhao et al., 2007; Erental et al., 2008; Li and Rollins, 2010; Schamber et al., 2010). A specific class of fungal transcription factors Ste12 (Ste12-like), downstream of MAPK, has emerged as a major component inducing appropriate adaptive responses (Wong Sak Hoi and Dumas, 2010). The Ste12 protein was first identified as a target of the Fus3 MAPK cascade regulating the mating and pseudohyphal/invasive growth pathways (Errede and Ammerer, 1989; Chou et al., 2006). In the present study, the SsSte12 was characterized in *S. sclerotiorum.* SmartBLAST represented a search result of the SsSte12 protein with two conserved motifs. One was the N-terminally located homeodomain-like motif, Ste, which was involved in DNA binding (Yuan and Fields, 1991), while the other one was tandemly arranged C_2_H_2_ zinc finger domain at the C-terminal, which was similar with other filamentous ascomycetes but differed from yeast Ste12 (Schamber et al., 2010). Phylogenetic analysis of SsSte12 and other Ste12-like proteins from Leotiomycetes and Sordariomycetes fungi demonstrated that SsSte12 encodes a homolog to *B. cinerea* Ste12 transcription factors and Ste12-like proteins of other ascomycetes (**Figure 1**).

In *S. sclerotiorum*, RNAi knockdowns could be employed in the loss-of-function of target genes (Kim et al., 2011; Fan et al., 2017; Li et al., 2018). In the present study, to avoid the potentially biased result caused by RNAi technique in this notorious fungus, we designed two target sites in the coding sequence of *SsSte12* and obtained four independent RNAi mutants for each target. We also included on empty vector transformant as control for the subsequent functional analysis assays. Besides, we compared the transcript accumulations in different strains, including the WT, EV, and RNAi strains, to demonstrate that the *SsSte12* was efficiently silenced in our experiments (**Figure 2**). Thus, the function of these eight SsSte2-silenced lines could be assessed based on the distinct trends, compared to the control strains (UF1 and pSD-EV).

As one of the key downstream targets of the MAPK signaling pathway, Ste12 acts as a central node in both mating and invasive growth response (Rispail and Di Pietro, 2010). Interestingly, Ste12 and Ste12-like proteins are also vital for pathogenesis in other animal and plant pathogens tested to date (Wong Sak Hoi and Dumas, 2010). The disruption of the *STE12* gene in *Cryptococcus gattii* led to a marked decrease of virulence in mouse model (Ren et al., 2006). In plant pathogens, *B. cinerea* mutants, lacking Ste12, showed delayed infection on plants as a result of low penetration efficiency (Schamber et al., 2010). In *Setosphaeria turcica* and *Magnaporthe grisea*, Ste12 was also required for the pathogenicity by regulating the appressorium development and penetration (Park et al., 2004; Gu et al., 2014). Given these positive roles of Ste12 orthologs in infection, we hypothesized that silencing *SsSte12* would cause phenotypes associated with appressorium development and pathogenicity. The current results showed that although RNAi mutants resulted in less quantity of compound appressoria, the appressorium formation was not affected. Consequently, the virulence was largely impaired when the expression of *SsSte12* was silenced in these RNAi mutants. Conversely, the inoculation on the wounded leaves result indicated that attenuated pathogenicity was due to fewer compound appressoria and impaired mycelial growth that were responsible for the virulence defects in these RNAi mutants. Similarly, the *B. cinerea Ste12* mutants also exhibited normal conidia germination and ability to invade and colonize the host tissue but delayed infection, which might be attributed to the inability to achieve appropriate cytoskeleton reorganization (Schamber et al., 2010). Thus, SsSte12 might stringently regulate less developed appressoria and exert no discernible role in regulating pathogenicity post-penetration, whereas in *B. cinerea*, the orthologous gene might only affect the highly differentiated appressoria that delay the infection.

Various phenotypes would also be observed in vegetative growth and sclerotia development as phenotypic effects on both sclerotium and compound appressorium development are a common finding in *S. sclerotiorum* (Jurick and Rollins, 2007; Li et al., 2012, 2017). Moreover, understanding these phenotypes has been under intensive focus to gain insights into the mechanisms underlying the MAPK pathway that connects the sclerotia development and pathogenicity. In this study, all *SsSte12*-silenced mutants showed similar phenotypes. Small-sized mature sclerotia were prominent features of the SsSte12-silenced mutants, and the vegetative growth of *S. sclerotiorum* was reduced as indicated by abnormal hyphal branching patterns and slow radial expansion. Similarly, the *N. crassa Ste12*-disrupted mutant showed a severe reduction in the vegetative growth and failure in developing protoperithecia (Li et al., 2005). However, in *B. cinerea* and *Cryphonectria parasitica*, loss-of-function mutants for *Ste12* exhibited loss of female fertility (Deng et al., 2006; Schamber et al., 2010) without vegetative growth phenotypes. Nevertheless, the function of Ste12p in female fertility is not a general feature, and a specific role of Ste12p or Ste12-like proteins in sexual development cannot be ascribed for all fungal species (Wong Sak Hoi and Dumas, 2010). Thus, the current results demonstrated that *SsSte12* could regulate the vegetative growth and sclerotial development in *S. sclerotiorum*, although this might not be the case for *B. cinerea*.

In *S. cerevisiae* cells, Ste12 interacts with Mcm1 proteins (Johnson, 1995), in parallel to the case of that SmSte12 associates with the SmMcm1 in *Sordaria macrospora* (Nolting and Pöggeler, 2006). We predicted the potential interactions between SsSte12 and SsMcm1 (**Supplementary Figure S2**) and this interaction was confirmed by Y2H and BiFC assays (**Figure 7**), suggesting a conserved interaction relationship between Ste12 and MCM1 proteins. BiFC data showed that SsSte12 interacts with SsMcm1 in the nucleus, implying their functions as transcription factors. A previous study showed Ste12 functions as a common regulator binding to numerous gene promoter regions (Borneman et al., 2007). In our study, the result showed that *SsMcm1* expression was independent of the regulation of *SsSte12* as revealed by detecting transcript accumulation of *SsMcm1* by qRT-PCR in *SsSte12* RNAi mutants. In this case, SsMcm1 might play a role in tethering SsSte12, allowing the transcriptional activation of a-specific target genes, which do not contain PREs in their promoters (Wong Sak Hoi and Dumas, 2010). Together with our previous study showing that SsMcm1 was essential for the growth and virulence of *S. sclerotiorum* (Qu et al., 2014) and other reported cases on the function of MCM1 on development and virulence (Zhou et al., 2011), we hypothesize that the SsSte12-SsMcm1 interaction might be involved in the regulation of a set of target genes encoding vegetative development, sclerotia development, and appressoria quantity traits in *S. sclerotiorum*. However, the complex regulatory network of SsSte12 protein and a complete perspective of the targets genes regulated by SsSte12 or SsSte12/SsMcm1 complex is yet to be elucidated.

## Conclusion

The *SsSte12* gene from *S. sclerotiorum* was characterized as an ortholog of the Ste12 C_2_H_2_-type zinc finger transcription factor. The present study provided evidence, which was in agreement with other fungal Ste12 orthologs such that SsSte12 played a critical role in regulating the development and pathogenicity. RNAi mutants of *SsSte12* resulted in phenotypes of delayed vegetative growth and formed small-sized sclerotia and attenuated pathogenicity on unwounded hosts as less developed compound appressoria. Together with high *SsSte12* gene transcript accumulation in the early development of *S. sclerotiorum*, the current results demonstrated that SsSte12 functions in vegetative mycelial growth, sclerotia development, and compound appressoria formation. The SsSte12-SsMcm1 interaction might play a critical role in the regulation of genes encoding these traits in *S. sclerotiorum*. However, further studies would assist in elucidating the signaling pathway and predicting the target genes of this class of transcriptional factors, and these processes at a molecular level are of concern for a better understanding of the developmental regulation and other filamentous fungi.

## Author Contributions

TX and JTL performed the experiments. JTL and BY analyzed the data and wrote the manuscript. YZ and HP conceived the study and provided funding. LL, XZ, and JLL provided technical support. All authors commented on the manuscript.

## Conflict of Interest Statement

The authors declare that the research was conducted in the absence of any commercial or financial relationships that could be construed as a potential conflict of interest.

## Footnotes

1 http://www.ncbi.nlm.nih.gov/

2 http://blast.ncbi.nlm.nih.gov/smartblast/

3 https://imagej.nih.gov/ij/

## Supplementary Material

FIGURE S1 Silencing *SsSte12* attenuated pathogenecity on hosts. Symptom development on detached tomato leaves were photographed by 2 DAI using mycelia-colonized PDA agar plugs.

FIGURE S2 Interaction proteins prediction of Ste12. **(A)** Ste12 of Yeast interaction network visualized by STRING. Network edges represent protein–protein associations, and line shape indicates the predicted mode of action. **(B)** Gene co-occurrence of these five interacted proteins with Ste12 in different organisms. KSS1, FUS3, and MCM1 proteins identified from Yeast system keep high conservation in *S. sclerotinia*.

TABLE S1 Primers used in this research.
