# Supplementary figures and images for: Transcription Factor SsSte12 Was Involved in Mycelium Growth and Development in Sclerotinia sclerotiorum
Source: Front Microbiol. 2018 Oct 17;9:2476. doi: 10.3389/fmicb.2018.02476 (PMC6200020; doi:10.3389/fmicb.2018.02476)

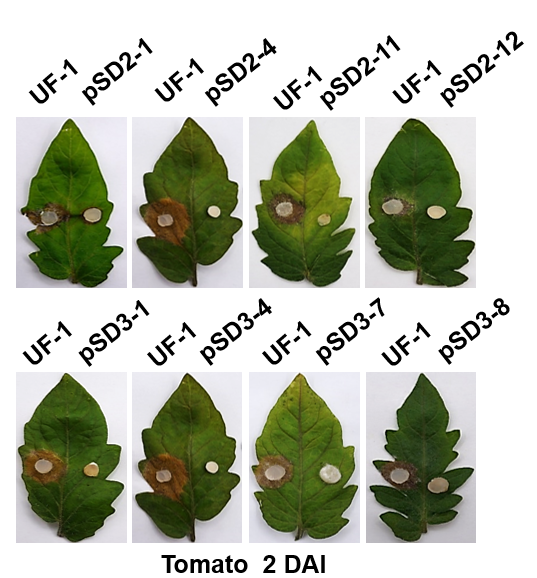

Supplement: FIGURE S1 — Silencing SsSte12 attenuated pathogenecity on hosts. Symptom development on detached tomato leaves were photographed by 2 DAI using mycelia-colonized PDA agar plugs. [file Image_1.TIF]

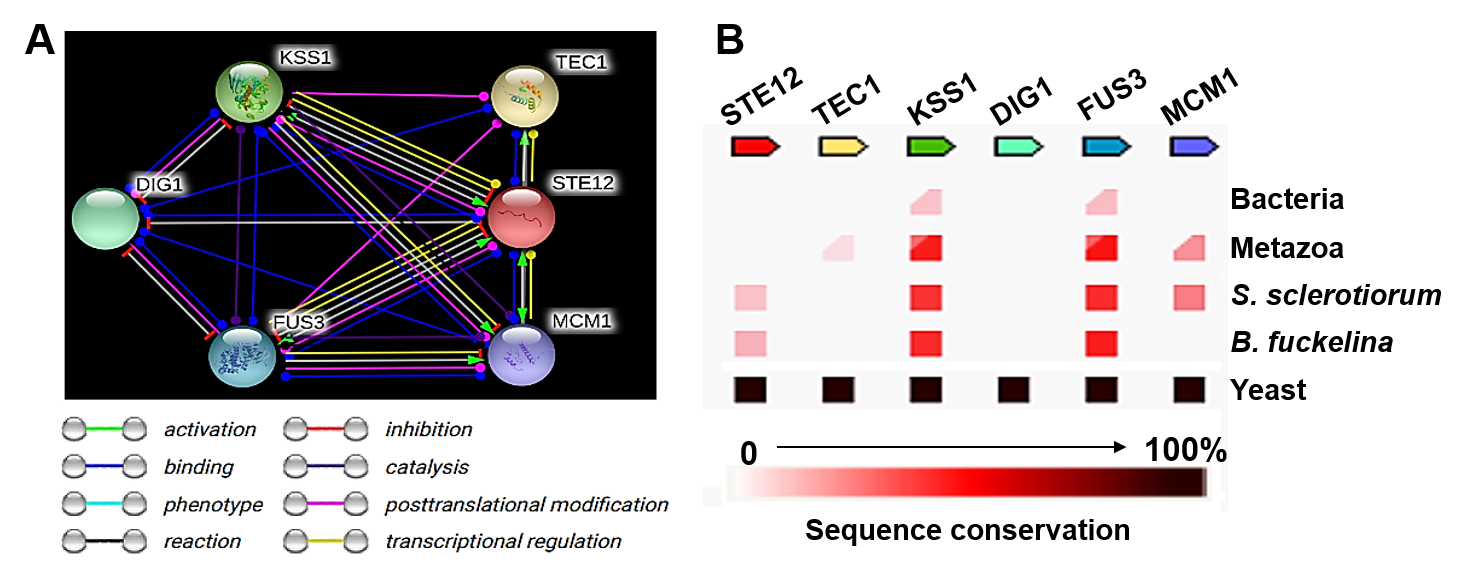

Supplement: FIGURE S2 — Interaction proteins prediction of Ste12. (A) Ste12 of Yeast interaction network visualized by STRING. Network edges represent protein–protein associations, and line shape indicates the predicted mode of action. (B) Gene co-occurrence of these five interacted proteins with Ste12 in different organisms. KSS1, FUS3, and MCM1 proteins identified from Yeast system keep high conservation in S. sclerotinia. [file Image_2.TIF]
