# Supplementary material for: Transcription Factor SsSte12 Was Involved in Mycelium Growth and Development in Sclerotinia sclerotiorum
Source: Front Microbiol. 2018 Oct 17;9:2476. doi: 10.3389/fmicb.2018.02476 (PMC6200020; doi:10.3389/fmicb.2018.02476)
Supplement: TABLE S1 — Primers used in this research. [file Table_1.DOCX]

Table S.1 Primers used in this research

| Primer name | Sequence |
| --- | --- |
| *SsSte12*-F  *SsSte12*-R | 5’-ATGGCTCCTCCACAAAAACCAGAGA-3’  5’-CTATATTGGTTGTTGCATGAGTTGA-3’ |
| pSD2-*SsSte12*-F  pSD2-*SsSte12*-R  pSD3-*SsSte12*-F | 5’-CCCAAGCTTCCAGAATGGTGTCCCGATAG-3’  5’-CCCAAGCTTCCAGAATGGTGTCCCGATAG-3’  5’-CCCAAGCTTGAAGATGGGACAGGAAGC-3’ |
| pSD3-*SsSte12*-R  G418-F  G418-R | 5’-CCCAAGCTTCATTTGTGGTTGCTGCATCGTC-3’  5’-TGTCCGGTGCCCTGAATGAACT-3’  5’-GCCGCCAAGCTCTTCAGCAATAT-3’ |
| pGBKT7-*SsSte12*-F | 5’-GCCATGGAGGCCGAATTCCCAATGGCTCCTCCACAAAAACC-3’ |
| pGBKT7-*SsSte12*-R  pGBKT7-*SsMcm1*-F  pGBKT7-*SsMcm1*-R  pGADT7-*SsMcm1*-F  pGADT7-*SsMcm1*-R | 5’-CTGCAGGTCGACGGATCCCCTTCTATATTGGTTGTTGCATGAGTTGA-3’  5’-GGAATTCATGGCCGATATCACAG-3’  5’-GCGTCGACTTATGATTGATGTGC-3’  5’-TAATACGACTCACTATAGGG-3’  5’-AGATGGTGCACGATGCACAG-3’ |
| pGADT7-*SsSte12*-F | 5’-GAGGCCAGTGAATTCCACCCAATGGCTCCTCCACAAAAACC-3’ |
| pGADT7-*SsSte12-*R | 5’-TCCCGTATCGATGCCCACCCTTCTATATTGGTTGTTGCATGAGTTGA-3’ |
| cYFP-*SsSte12*-F | 5’-GTCGACGGTACCGCGGGCCCATGGCTCCTCCACAAAAACC-3’ |
| cYFP-*SsSte12*-R  nYFP-*SsMcm1*-F  nYFP-*SsMcm1*-F  qRT-*SsMcm1*-F  qRT-*SsMcm1*-R  qRT-*SsSte12*-F  qRT-Ss*Ste12*-R | 5’-TTGCTCACCATCAGGATCCCTATTGGTTGTTGCATGAGTTGATGT-3’  5’-AGCTCAAGCTTCGAATTCATGGCCGATATCACAG-3’  5’-CCGTCGACTGCAGAATTCTGATTGATGTGCTTGC-3’  5’-CCAAGTCCTCCTCCTAGTCGTATCC-3’  5’-GGCTCCTCTGGCGATTCAACTG-3’  5’-CGAGCGATAGATTCAATGCCTCCTC-3’  5’-CTGCTGTTGATGATGTTGATGTGGTTG-3’ |
| *Actin*-F  *Actin*-R | 5’-GAATGTGTAAGGCCGGTTTCGC-3’  5’-CATCCCAGTTGGTGACGACACC-3’ |
